# Towards a dynamic clamp for neuro-chemical modalities

**DOI:** 10.1186/1471-2202-14-S1-P232

**Published:** 2013-07-08

**Authors:** Alexander G Dimitrov, Wei Xue, Jie Xu, Catalina Maria Rivera, Gan Yu, Jihen Zhao, Hyuck-Jin Kwon

**Affiliations:** 1 Department of Mathematics, Washington State University, Vancouver, WA 98686, USA; 2 Department of Mechanical Engineering, Washington State University, Vancouver, WA 98686, USA

## 

The classic dynamic clamp technique uses a real-time electrical interface between living cells and neural simulations in order to investigate hypotheses about neural function and structure [3]. However, it has two major drawback [2]: the electrodes can clamp electrically only a small section of a cell, and hence frequently do not control the behavior of whole cells; and all control to-date has been concentrated on the electric properties of neurons, neglecting their chemical state. As noted in [2], the latter has been done by necessity, since until recently registering or controlling the chemical state of either neurons or the chemical environment in which they reside has been essentially impossible with the level of precision needed to simulate the appropriate dynamics of the various extracellular chemical players. In this manuscript we present an expansion of the dynamic clamp method to include simulation and control of the effects of a variety of chemicals associated with neural signaling. To achieve that, we use the emergent discipline of microfluidics [4], which deals specifically with the behavior, precise control and manipulation of fluids at the microscale. We use a novel combination of microfluidic and nanosensor technology to add sensing and control of chemical concentrations to the dynamic clamp technique. Specifically, we use a microfluidic chip to generate distinct chemical concentration gradients (ions or neuromodulators), register the concentrations with embedded nanosensors, and use the processed signals as an input to simulations of a neural cell. The ultimate goal of this project is to close the loop, and provide control signals to the microfluidic lab to mimic the interaction of the simulated cell with other cells in its chemical environment, by modifying the chemical concentrations in the microfluidic lab environment to reflect simulated outputs of the model neurons. Here we used Hodgkin-Huxley type neurons, $C\frac{dV}{dt}=I_{o}+I_{HH}+I_{Ca}$with added calcium-gated currents,

$$I_{Ca}=m_{\infty }{\left(V\right)}^{2}hP_{max}\frac{z^{2}F^{2}}{RT}V\left(\frac{{\left[Ca\right]}_{in}-{\left[Ca\right]}_{out}e^{\frac{-zVF}{RT}}}{1-e^{\frac{-zVF}{RT}}}\right)$$

In order to predict the distribution of specific chemicals inside a microfluidic chamber, 2-D Computational Fluid Dynamics technique was used. In this study, the commercial software FLUENT® 6.3 was used to build the computational domain, the models for a microfluidic mixer and a neuron chamber by using finite element methods.

**Figure 1:**
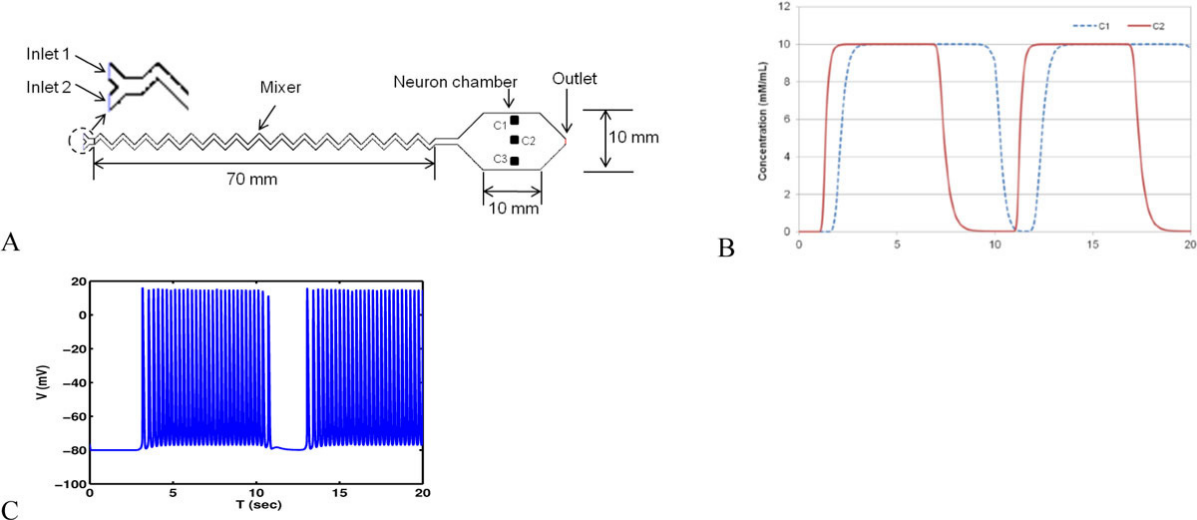
Shows the simulated microfluidic device (A) and simulated square pulse flow at pts C1 and C2 (B). The responses of the model neuron to *changes in Ca++ concentration *are shown in panel C. The device in (A) was realized physically, using standard lithography and bonding methods as described in [1]. The Ca++ concentration was recorded and the record was used as an input to the same model neuron, with results indistinguishable from (C).
